# Understanding the moderating role of gender in physical activity enjoyment and mental well-being: evidence from university students’ campus recreation participation

**DOI:** 10.3389/fpubh.2025.1679353

**Published:** 2025-10-07

**Authors:** Omur Fatih Karakullukcu, Cihan Ayhan, Ferhat Guder, Laurentiu-Gabriel Talaghir, Dumitru Marius Cosoreanu, Cristina Corina Bentea

**Affiliations:** ^1^Ministry of National Education, Ankara, Türkiye; ^2^Faculty of Sport Sciences, Sakarya University of Applied Sciences, Sakarya, Türkiye; ^3^Faculty of Sport Sciences, Bayburt University, Bayburt, Türkiye; ^4^Faculty of Physical Education and Sport, Dunarea de Jos University of Galati, Galati, Romania

**Keywords:** campus recreation, mental well-being, outdoor recreation, physical activity, recreational benefit

## Abstract

**Background:**

Participation in physical activity is considered an important factor not only for individuals’ physical health but also for their psychological well-being. Developing physical activity habits, especially among university-aged individuals, plays a critical role in long-term health outcomes. In this context, the impact of physical activity enjoyment on individuals’ mental well-being has become a topic of interest. However, the moderating role of individual differences, particularly gender, in this relationship has not yet been sufficiently clarified. The purpose of this study was to examine the relationship between physical activity enjoyment and mental well-being among university students and to reveal the moderating effect of gender on this relationship.

**Method:**

The research was conducted using a relational screening model. The study group consisted of 392 university students (261 male and 131 female; mean age = 22.95 ± 2.58) from Sakarya University of Applied Sciences who participated in campus recreation activities. Data were collected via a face-to-face survey using the Physical Activity Enjoyment Scale and the Mental Well-being Scale. Descriptive statistics, Pearson correlation analysis, and regression-based moderation analysis using PROCESS Macro Model 1 were used in the statistical analysis.

**Results:**

The level of enjoyment of physical activity was found to have a significant positive effect on mental well-being (*r* = 0.427, *p* = 0.001; *F* = 50.388, *p* < 0.001; *R*^2^ = 0.280). Moderation analysis further showed that gender significantly moderated this relationship (Δ*R*^2^ = 0.049; *p* < 0.001). The effect of enjoyment on mental well-being was significant for both males (*β* = 0.2693; S.E. = 0.0348; *p* < 0.001; 95% CI [0.2010, 0.3377]) and females (*β* = 0.7913; S.E. = 0.0949; *p* < 0.001; 95% CI [0.6047, 0.9780]), with the effect being stronger among females.

**Conclusion:**

The findings show that considering gender-sensitive approaches in physical activity programs to be implemented on university campuses has a potential impact on improving the psychological well-being of students.

## 1 Introduction

In Turkey, 37.6% of individuals aged 19 and over do not reach the physical activity level recommended by the World Health Organization for a healthy life (1). Furthermore, this situation becomes a more pronounced public health problem with increasing age (2). Inadequate physical activity is a significant risk factor for chronic diseases, including obesity, cardiovascular problems, type 2 diabetes, hypertension, several cancers, and mental health disorders such as depression and anxiety (3–5). Encouraging participation is therefore critical for both physical and mental health (6–8), and developing habits during young adulthood can be decisive for long-term outcomes. In addition to physical health, recent studies underline that the psychological mechanisms underlying participation, such as enjoyment and motivation, must be examined to explain why some individuals sustain activity while others discontinue (9).

Physical activity opportunities offered on university campuses play a significant role, particularly because lifestyle habits acquired in early adulthood have the potential to be carried into later life (10–12). Campus recreation programs are also associated with benefits such as stress reduction, holistic health, physical strength, and academic success (13–16). The enjoyment of these activities increases participation, boosts motivation, and helps them become long-term habits (17). Enjoyment is considered a critical affective determinant that predicts whether individuals transition from short-term participation to lifelong adherence, particularly during university years when autonomy increases but stress levels are also high (10). This suggests that, beyond general health outcomes, identifying the specific psychological and social mechanisms underlying activity participation is crucial for addressing concrete research gaps.

Physical activity enjoyment is defined as an individual’s positive emotional experience as a result of physical activities, associated with feelings such as pleasure, joy, and fun. It is considered an important determinant of physical activity participation, especially for children and adolescents (18–23). Since positive affective experiences in physical activity are closely linked to broader psychological functioning, examining them alongside mental well-being provides a more comprehensive understanding of individuals’ health and quality of life.

The concept of mental well-being is defined as the individual being aware of his/her own potential, being able to cope with the stress in his/her life, being a productive and useful individual and contributing to society; it is also explained as the individual being satisfied with his/her emotional, cognitive and environmental states and having inner peace in this context (24, 25).

Men and women may enjoy physical activities differently, and this can have different effects on their mental health (26, 27). It has been reported that male students generally gravitate towards competitive and high-intensity activities, while female students prefer activities focused on aesthetics, social interaction, and health (28). However, gender cannot be reduced to a fixed binary category. Instead, it is increasingly conceptualized as a socio-cultural construct shaped by psychological processes, cultural expectations, and institutional practices that influence how individuals perceive and experience physical activity (29, 30). These gender differences may moderate the relationship between physical activity enjoyment and mental well-being by altering the direction and strength of this relationship. In this context, the impact of physical activity on individuals is not limited to physical competence; it also contributes to an individual’s mental well-being. Further, Gender Schema Theory (GST) posits that gendered cognitive schemata shape how individuals interpret physical activity experiences, influencing both interpretation and emotional response in ways that go beyond binary categorizations (31).

Self-Determination Theory (SDT) provides a valuable theoretical framework for understanding individuals’ motivation toward physical activity. According to Ryan and Deci (32), intrinsic motivation refers to engaging in an activity for the inherent enjoyment and satisfaction it provides. The theory proposes that the fulfillment of three basic psychological needs—competence, autonomy, and relatedness—fosters intrinsic motivation, which subsequently enhances participation and well-being. In campus recreation settings, meeting these needs can directly influence students’ enjoyment of physical activity. Students who choose activities freely, perceive themselves as competent, and experience social connectedness are more likely to report higher levels of enjoyment and mental well-being. This, in turn, enhances the quality of their motivation and supports sustained participation over time.

Furthermore, Ryan and Deci (33) conceptualize motivation not as a simple dichotomy of intrinsic versus extrinsic, but as a continuum of self-determination. This continuum ranges from amotivation, representing the lowest level of autonomy, through external, introjected, identified, and integrated regulation, ultimately culminating in intrinsic motivation. Such a perspective highlights that students’ reasons for engaging in physical activity differ in the extent of autonomy they embody, and these differences shape how enjoyment translates into well-being. For instance, the experience of a student who participates only due to external pressure differs markedly from that of a student who exercises because it aligns with personal values or self-development goals.

Evidence from Teixeira et al. (9) further demonstrates that SDT offers a robust theoretical basis for explaining the role of enjoyment in sustaining long-term participation in physical activity. Accordingly, campus recreation programs that provide autonomy support, enhance competence, and foster social relatedness are more likely to promote not only short-term enjoyment but also the adoption of physical activity as a lasting lifestyle. Thus, SDT serves as a comprehensive framework to explain the link between physical activity enjoyment and mental well-being (9, 32, 33). In this framework, Bem’s (34) GST posits that individuals construct schemas related to gender roles, which over time shape their self-concept. According to Bem, people internalize the culturally defined characteristics of masculinity and femininity and evaluate themselves against these standards. This process influences how individuals judge which activities are appropriate for them and which situations they find more enjoyable. Gender schemas act as cognitive filters that affect the way environmental stimuli are processed and simultaneously shape preferences for physical activity and the psychological satisfaction derived from such activities. Recent empirical evidence supports this view, indicating that gender influences not only behavioral preferences but also the cognitive mechanisms underlying activity-related decision-making (35).

For instance, the tendency of male students to enjoy competitive activities more in campus recreation, while female students gravitate toward aesthetic and socially interactive activities, can be explained by gender schemas formed in childhood and reinforced over time. As Starr and Zurbriggen (29) highlight, GST extends beyond behavioral choices; it also shapes cognitive processing, memory, perception, and decision-making. Individuals are more likely to encode and recall gender-congruent information, and to make self-judgments more readily when aligned with their gender schemas. Similarly, studies on campus recreation environments have revealed that female students often experience lower comfort levels, particularly in weight-training spaces, reflecting the influence of schema-driven expectations and social norms (30).

Importantly, GST is not rooted in essentialist biological differences but in cultural constructions that are internalized through socialization and reinforced by societal institutions. From this perspective, avoiding essentialist assumptions is critical; gendered experiences in physical activity should be interpreted as socially constructed rather than biologically determined. Within this perspective, gender is not treated merely as a binary variable but as a socio-cultural process shaping how individuals experience physical activity and the mental well-being derived from it. Thus, GST provides a valuable conceptual lens for understanding why the relationship between physical activity enjoyment and mental well-being may differ between male and female students. At this point, the interaction arising from the intersection of SDT and GST is noteworthy. SDT argues that individuals experience higher levels of enjoyment and mental well-being when they participate in activities motivated by intrinsic motivation, while GST suggests that individuals’ motivations and perceived preferences for these activities are socially shaped. In this context, individuals’ intrinsic motivation levels interact not only with their psychological needs but also with gender-based cognitive structures. How men and women enjoy physical activities may vary depending on both the level of meeting their internal needs and socially internalized gender roles. This explains how the intrinsic motivation mechanism offered by SDT is shaped by GSs and why gender can be a moderating variable (32, 34). Therefore, this theoretical framework contributes to understanding why gender may play a moderating role in the relationship between physical activity enjoyment and mental well-being.

In recent years, the concept of physical activity in campus recreation activities has become an important research area in sports sciences (36–38), and physical activity is effective in preventing life-threatening diseases (39–42). Most of these studies have primarily focused on physical health outcomes and disease prevention, with relatively less attention given to psychological and affective dimensions. However, limited studies are addressing the relationship between physical activity and mental well-being (43, 44), these works have largely concentrated on general associations without investigating contextual factors such as enjoyment or socio-demographic moderators. However, no studies have examined the moderating role of gender in the relationship between physical activity enjoyment in campus recreation activities and mental well-being. Therefore, the lack of knowledge in this area indicates that it is an area that needs to be supplemented with new studies. In this context, understanding how the relationship between physical activity enjoyment and mental well-being differs across gender is becoming increasingly important in the sports sciences literature. Considering the dynamics of this differentiation, a deep understanding of the moderating role of gender and its effects on the well-being of university students is important to fill the gaps in the literature.

This study aims to understand the moderating role of gender in the relationship between physical activity enjoyment and mental well-being in campus recreation activities. This research will contribute to filling a gap in the literature and guide future research. Practically, the study findings may guide the development of gender-sensitive physical activity programs on university campuses and contribute to the development of strategies to enhance students’ mental well-being. Thus, they may contribute to the design of more inclusive, effective, and sustainable physical activity environments at universities. Based on these theoretical and empirical foundations, the hypotheses to be tested in this study are presented below.

*H_1_:* Enjoyment of physical activity significantly and positively predicts individuals' mental well-being.

*H_2_:* Gender has a significant moderating effect on the relationship between enjoyment of physical activity and mental well-being.

## 2 Method

### 2.1 Research model

**Figure 1:**
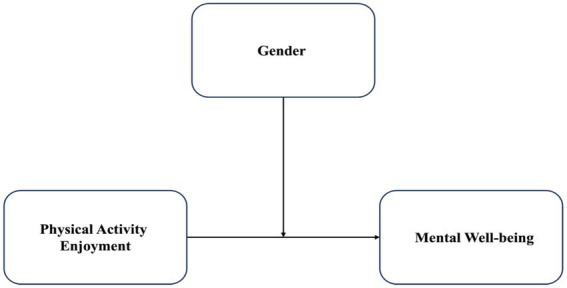
Research model.

This research was designed using the correlational screening model, a quantitative research method. The correlational screening model is an approach that aims to examine the relationships between variables without intervening in the current situation (45). The study examined the relationship between physical activity enjoyment and mental well-being, and the moderating role of gender in this relationship was tested. The research model is presented in Figure 1.

### 2.2 Study sample

The study sample consisted of individuals who participated in campus recreation activities organized on the Sakarya University of Applied Sciences campus. Participants were students who regularly took part in various campus recreation activities, including outdoor sports, fitness, football, basketball, and tennis. Within the scope of the research, participants were recruited using a convenience sampling method. The inclusion criterion for the study was that individuals had participated in at least one recreational activity organized within the university within the last month. After eliminating incomplete or incorrectly completed forms from a total of 400 individuals, data from 392 participants were included in the study. Of the participants, 261 were male (66.6%) and 131 were female (33.4%). The mean age was 28.36 ± 7.31 for males and 27.46 ± 5.96 for females, while the overall mean age was 22.95 ± 2.58, with the age distribution ranging from 18 to 29. Regarding weekly frequency of participation, 151 students (38.5%) reported attending once per week, 166 students (42.3%) twice per week, and 75 students (19.1%) three or more times per week. In terms of the purpose of participation, 82 students (20.9%) engaged primarily for physical health, 228 (58.2%) for mental health, and 82 (20.9%) for socialization. According to Sekaran (46), when the population exceeds 10,000,000, a minimum of 384 participants is considered sufficient, and sample sizes between 30 and 500 are generally appropriate. Therefore, the inclusion of 392 participants in this study meets these criteria and enhances the statistical power and generalizability of the findings. Descriptive statistics for the participants are given in Table 1.

**Table 1:** Demographic characteristics of the participants.

Variable	Category	Frequency	(%)
Gender	Male	261	66.6
	Female	131	33.4
Participation (weekly frequency)	1 time	151	38.5
	2 times	166	42.3
	3 times or more	75	19.1
Purpose of participation	Physical health	82	20.9
	Mental health	228	58.2
	Socialization	82	20.9
Age	**Category**	**Mean**	**S.D.**
	Male	28.36	7.31
	Female	27.46	5.96
	Overall	22.95	2.58

### 2.3 Data collection tools

All participants were included in the study voluntarily, were informed in advance about the purpose of the study, and their consent was obtained following ethical principles. To collect data in the study, the Personal Information Form prepared by the researcher to determine the demographic characteristics of the participants, as well as the Physical Activity Enjoyment Scale and the Mental Well-being Scale, were used. Data were collected via a face-to-face survey, and each participant responded to the questions for an average of 5–7 min.

#### 2.3.1 Personal information form

To determine the moderating effect of gender and to provide information about the average age of the sample to be suitable for the purpose of the research, only questions about the age and gender of the participants, as well as their weekly frequency of participation in campus recreational activities and their purpose of participation, were included in the Personal Information Form.

#### 2.3.2 Physical activity enjoyment scale

To measure the level of enjoyment from physical activity, the scale developed by Mullen et al. (47) and adapted into the Turkish language by Özkurt et al. (48) was used. The physical activity enjoyment scale has a single-factor structure and consists of eight items. Participants responded to these items on a 7-point Likert-type rating scale ranging from 1 (Strongly Disagree) to 7 (Strongly Agree). High mean scores indicate a high level of enjoyment from physical activities, while low mean scores indicate a low level of enjoyment. In the adaptation of the scale to Turkish culture, the Cronbach’s *α* reliability coefficient was reported as 0.955. In the current study, Cronbach’s *α* was calculated as 0.939. This result demonstrates that the scale has a high level of internal consistency and reliability (49).

#### 2.3.3 Mental well-being scale

To measure general mental well-being, the Warwick-Edinburgh mental well-being scale was developed by Tennant et al. (50) and adapted into the Turkish language by Keldal (51) was used. The mental well-being scale has a single-factor structure and consists of 14 items. Items were answered on a 5-point Likert-type scale ranging from 1 (Never) to 5 (Always) and do not include reverse-scored items. Higher scores indicate a higher level of mental well-being. In the Turkish adaptation study, Cronbach’s *α* reliability coefficient was reported as 0.92. In the present study, Cronbach’s *α* was found to be 0.885. These findings demonstrate that the scale has adequate internal consistency (49).

### 2.4 Ethical approval

This research was ethically approved by the Sakarya University of Applied Sciences Ethics Committee with decision number 39 taken at the meeting number 56 dated 07.05.2025 (Decision Number: 56/39).

### 2.5 Statistical analysis

The data obtained in the study were analyzed using the IBM SPSS Statistics 25.0 program and the PROCESS Macro v4.0 plug-in (52). Before the analysis, preliminary checks were conducted to assess the suitability of the data for statistical analysis. To determine whether the normal distribution assumption was met, the skewness and kurtosis values of the variables were examined between −2 and +2 (53). As a result of this evaluation, it was determined that the data showed a normal distribution, and parametric tests were applicable. During the data analysis process, firstly, descriptive statistics of the measurement tools used in the study, reliability coefficients (Cronbach’s *α*), and Pearson correlation analysis were conducted to determine the level of relationships between the variables. Then, in line with the main objective of the study, the moderator role of gender in the relationship between enjoyment from physical activity and mental well-being was examined. For this purpose, a regression-based moderation analysis was applied using the Model 1 option of PROCESS Macro developed by Hayes (52). In the model, physical activity enjoyment was defined as the independent variable, mental well-being as the dependent variable, and gender as the moderator variable. Gender, used as the moderator variable in the moderation analysis, was converted to a dummy variable before the analysis (0 = male, 1 = female). The Bootstrap method was used in the analysis, and significance was assessed with a 95% confidence interval over 5,000 samples. The fact that the confidence intervals obtained in this method do not include the value zero indicates that the effects are significant (54, 55). In addition, a simple slope analysis was performed on the PROCESS Macro outputs to detail the findings of the moderation analysis. Within the scope of this analysis, the effect of the enjoyment variable on mental well-being was examined separately for each level of gender (male and female).

## 3 Results

When the analysis results in Table 2 were examined, it was determined that there is a positive, moderately statistically significant relationship between enjoying physical activity and mental well-being (*p* < 0.01).

**Table 2:** The relationship between enjoyment from physical activity and mental well-being.

Variable 1	Variable 2	*r*	*p*
Enjoyment	Mental well-being	0.427	0.001

When the analysis results were examined, the regression model testing the relationship between physical activity enjoyment and mental well-being was found to be significant (*F* = 50.388; *p* < 0.001), and the model’s explanatory power was 28.0% (*R*^2^ = 0.280). The physical activity enjoyment variable was found to have a significant and positive effect on mental well-being (*β* = 0.2693; *p* < 0.001). The physical activity enjoyment variable was found to have a significant and positive effect on mental well-being (*β* = 0.2693; *p* < 0.001). Furthermore, the main effect of gender on mental well-being was statistically significant (*β* = −3.5410; *p* < 0.001). This result indicates that female individuals have lower levels of mental well-being than male individuals. The inclusion of the interaction term (enjoyment × gender) in the model significantly improved the model (Δ*R*^2^ = 0.0494; *p* < 0.001). This result indicates that gender has a moderating effect on the relationship between enjoyment and mental well-being. The regression coefficient for this interaction was also statistically significant (*β* = 0.5220; *p* < 0.001). This finding suggests that the effect of enjoyment on mental well-being differs by gender (Table 3).

**Table 3:** Moderation analysis: gender as a moderator between enjoyment and mental well-being.

Model	*β*	S.E.	*t*	*p*	LLCI	ULCI
Constant	2.2677	0.2121	10.6929	<0.001	1.8507	2.6846
Enjoyment	0.2693	0.0348	7.7457	<0.001	0.2010	0.3377
Gender	−3.5410	0.6179	−5.7307	<0.001	−4.7559	−2.3262
Enjoyment × Gender	0.5220	0.1011	5.1628	<0.001	0.3232	0.7208
Model-related values	***R***	***R*** ^**2**^	***F***	***p***		
	0.5295	0.2804	50.388	0.001		

Δ*R*^2^: 0.0494; *p* < 0.001.

According to the moderation analysis, the effect of physical activity enjoyment on mental well-being differs significantly by gender. The effect of enjoyment on mental well-being was found to be statistically significant in males (*β* = 0.2693; S.E. = 0.0348; *p* < 0.001; 95% CI [0.2010; 0.3377]). This effect was higher in females (*β* = 0.7913; S.E. = 0.0949; *p* < 0.001; 95% CI [0.6047; 0.9780]) and was significantly higher. These findings suggest that increased enjoyment from physical activity positively impacts mental well-being in both genders, but this effect is stronger in women. Therefore, gender stands out as a moderating factor in the relationship between enjoyment of physical activity and mental well-being (Table 4).

**Table 4:** Conditional effects of enjoyment on mental well-being by gender.

Gender	Effect *β*	S.E.	*t*	*p*	LLCI	ULCI
Male (0)	0.2693	0.0348	7.7457	<0.001	0.2010	0.3377
Female (1)	0.7913	0.0949	8.3351	<0.001	0.6047	0.9780

**Figure 2:**
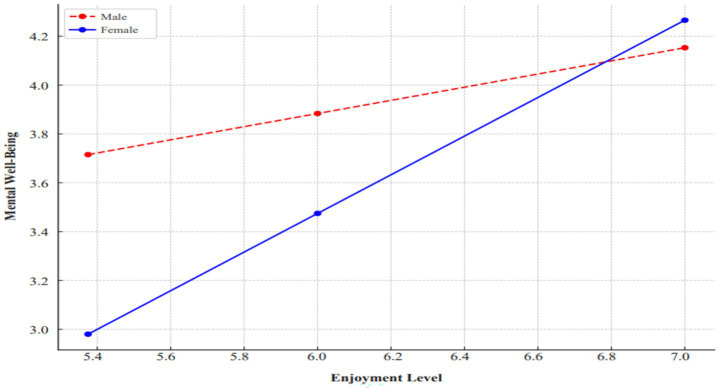
Interaction between enjoyment and mental well-being by gender.

Figure 2 visualizes the relationship between physical activity enjoyment and mental well-being by gender. Figure 2 shows that increasing enjoyment increases mental well-being in both gender groups. However, the slope of this relationship is steeper in women (blue line) than in men (red line). This finding suggests that the effect of enjoyment on mental well-being is stronger in women, indicating that gender significantly regulates this relationship (a moderator effect). Specifically, at lower levels of enjoyment, men have higher mental well-being scores, but as enjoyment increases, women’s mental well-being scores increase more rapidly. This is also supported by the significant interaction term obtained in the analyses (*β* = 0.5220; *p* < 0.001).

## 4 Discussion

In this study, the relationship between physical activity enjoyment and mental well-being in individuals participating in campus recreation activities was examined and it was also evaluated whether gender had a moderating role in this relationship. The findings were interpreted, compared with similar studies in the literature, and a comprehensive discussion of the results was presented. With a specific focus on the moderating role of gender. The discussion emphasizes how these findings fit within motivational and socio-cultural theoretical frameworks.

When the analysis results were examined, a moderately positive and statistically significant correlation was identified between physical activity enjoyment and mental well-being (*p* < 0.01). This outcome indicates that physical activity contributes not only to physical health but also to psychological well-being. A comprehensive meta-analysis by Singh et al. (56) demonstrated that physical activity exerts significant positive effects on depression, anxiety, and psychological stress across diverse populations. In parallel, previous research has consistently shown that physical activity enhances subjective well-being while reducing adverse psychological states such as depression and anxiety (57–59).

The present study provides an additional perspective by emphasizing the role of enjoyment, suggesting that beyond the frequency of participation, the affective experience of activity is a central determinant of psychological outcomes. Ekkekakis et al. (60) demonstrated that the pleasure experienced during physical activity significantly influences both psychological responses and long-term adherence. Consistent with this, Lewis et al. (61) reported that enjoyment was a stronger predictor of sustained physical activity behavior than self-efficacy. These findings highlight that enjoyment not only yields immediate affective benefits but also facilitates long-term participation, which is closely linked to improvements in mental well-being.

From a theoretical standpoint, SDT (62) posits that intrinsically motivated activities promote higher levels of psychological satisfaction. Empirical evidence supports this proposition: Lubans et al. (63) found that intrinsically motivating activities in young populations positively influenced cognitive and emotional health outcomes. Similarly, Zhang and Chen (64) underscored that physical activity enhances happiness across age groups, with such effects being closely tied to the emotional processes experienced during participation. Complementary findings by Ábrahám et al. (65) further indicated that the enjoyment of leisure activities, particularly those with social, intellectual, and introspective dimensions, makes a substantial contribution to psychological well-being. Collectively, these results reinforce the notion that the qualitative aspects of physical activity and the affective experiences they generate are crucial in explaining their impact on mental well-being.

When the relevant literature was examined, many studies supported the findings of this research. Eddolls et al. (66) report that physical activity and being fit were positively associated with mental health and quality of life in adolescents. Lindwall et al. (67) revealed that there is a two-way interaction between physical activity and depression and that this relationship is also valid for older individuals. Similarly, Hawker (68) reported the positive effects of physical activity on mental health in nursing students. A growing body of research has emphasized that physical activity plays a significant role not only in physiological outcomes but also in psychological gains, and that individual differences shape this effect (69–76). The consistency of the research results with the current literature indicates that campus recreational activities could be associated with improvements in students’ physical, emotional, and cognitive well-being; however, these interpretations should be made cautiously given the methodological limitations of the study. The study conducted by Liu et al. (77) revealed that physical activity has positive effects on health behaviors, mental health, and psychological resilience, and quality of life plays a significant regulatory role in these relationships. This finding reveals the multifaceted nature of physical activity and supports the relationship between enjoyment from physical activity and mental well-being, which is an important finding of our research.

The results of the analysis regarding the moderating role of gender revealed that the relationship between physical activity enjoyment and mental well-being differed significantly. In the regression analysis, it was observed that including the interaction term (Enjoyment × Gender) in the model significantly improved the model and increased explanatory power. While the effect of physical activity enjoyment on mental well-being was significant in male participants, this effect was observed to be higher in females than in males. When Figure 2 was examined, it was seen that as the level of enjoyment increased, mental well-being scores also increased in both genders, with a relatively steeper slope for women. This suggests that the association between enjoyment and mental well-being may be stronger among female participants. However, this interpretation should be approached cautiously, as factors such as differences in duration or type of physical activity participation, which were not controlled in this study, may also contribute to this pattern. This finding indicates that individuals’ emotional responses to physical activity may be shaped not only by personal differences but also by socially constructed gender roles. Previous studies suggest that men and women often engage in physical activities in distinct ways, which can influence both the enjoyment derived from participation and how this enjoyment is reflected in their mental well-being (26, 28). These findings may be interpreted within the frameworks of SDT (32) and GST (34), although neither theory was directly tested in this study. According to SDT, higher levels of intrinsic motivation are associated with greater enjoyment and, in turn, better mental well-being (62). From the perspective of GST, socially constructed gender schemas may influence perceptions of which activities are considered appropriate and how satisfaction is derived (34). In line with previous research, women may be more likely to view physical activity as a means of social connection, stress relief, or self-care, which could help explain why increases in enjoyment are more strongly associated with improvements in mental well-being among female participants.

Many studies in the relevant literature supported our research findings. A study conducted by Tian et al. (78) examined the mediating role of subjective well-being and the moderating effect of gender in the relationship between physical activity and anxiety. According to the research results, physical activity was significantly associated with subjective well-being and anxiety. Furthermore, while subjective well-being played a mediating role in the relationship between physical activity and anxiety, the gender variable had a moderating effect in this model. It was noted that the path coefficients, particularly in the direct impact between physical activity and anxiety, differed significantly between male and female participants. This finding demonstrates that the psychological results of physical activity may vary depending on an individual’s gender, and gender is an important variable to consider in the relationship between physical activity and psychological well-being. In an intervention study conducted by Medina et al. (79), it was suggested that the effects of moderate-to-vigorous exercise programs on anxiety sensitivity may vary by gender, and male participants were observed to benefit more from exercise. It was noted that men experienced a more significant decrease in anxiety sensitivity compared to women in the first week of the study, but this difference disappeared after the two-week intervention. This finding suggests that psychological responses to physical activity may differ based on gender in the short term. In the current study, a more significant increase in mental well-being was observed in women as their enjoyment of physical activity increased. Although Medina et al. (79) reported a faster response rate in men, both studies shared a common point that gender served as a decisive moderator. In other words, it suggests that psychological responses to physical activity may be shaped by gender-specific characteristics, social roles, and individual experiences. In the study conducted by Abós et al. (80), it was revealed that female students reported more negative motivational outcomes in response to internally controlling teaching behaviors compared to male students. This finding suggests that women’s psychological responses in physical activity contexts may be more strongly influenced by how the activity is perceived and delivered. These results are consistent with our findings, which indicate that increases in enjoyment of physical activity are more strongly associated with improvements in mental well-being among female participants.

## 5 Conclusion

This study highlights the significant role of physical activity enjoyment in enhancing individuals’ mental well-being, particularly in university students participating in campus recreation activities. The findings confirm that enjoyment is not only a motivational factor but also a crucial emotional component that contributes to psychological health. Furthermore, the moderating effect of gender indicates that this relationship differs between men and women, with women experiencing a greater positive impact on mental well-being as their enjoyment increases. These results suggest that both intrinsic motivational factors, as emphasized by SDT, and socially shaped perceptions related to gender, as outlined in GST, play an important role in shaping psychological outcomes in physical activity contexts.

### 5.1 Limitations and strengths of the study

As the sample of this study was drawn from a single university and selected through convenience sampling, the generalizability of the findings is limited. This study offers significant contributions to the literature by examining the relationship between physical activity enjoyment and mental well-being among university students participating in campus recreation activities, and the moderating role of gender in this relationship. However, the study has several limitations. First, the research data were obtained from a student sample from only one university and did not include participants from different university types (public and/or private) or sociocultural contexts. This limits the generalizability of the findings. Furthermore, because the data were collected through self-report, it was not possible to eliminate participants from influences such as social desirability bias or respondent bias. Furthermore, the study considered the gender variable as a binary (female–male) variable, excluding the cultural and psychosocial dimensions of gender. This situation has limited our ability to gain a more in-depth understanding of the impact of gender on individuals’ physical activity experiences. Another limitation of this study is that the duration of participants’ engagement in physical activity was not measured. Some individuals may have recently started participating in campus recreation activities, while others may have been active for a longer period. Such variability in the duration of participation could influence both the level of enjoyment and the perceived mental well-being, and thus may partly account for the observed differences between male and female participants. In addition, the age distribution of the participants (18–29 years) was relatively narrow, which may restrict the applicability of the results to other age groups. Finally, the higher number of male participants compared to female participants constitutes a limitation that should be taken into account when interpreting the findings. However, the robustness check conducted by equalizing group sizes showed that the results were consistent with the main analysis.

Despite these limitations, the study also has some strengths. In particular, its examination of the impact of physical activity enjoyment on mental well-being within the context of gender makes the study original and fills a gap in the literature. In this context, the study’s foundation in theoretical foundations such as SDT and GST allowed for a theoretical discussion of the relationships between variables. Furthermore, the inclusion of moderation analysis to test the moderating role of gender and the use of advanced statistical analysis techniques such as PROCESS macro strengthen the study’s methodological quality. Finally, obtaining findings that will guide practices to support the mental health of university students increases the practical value of the study as well as its theoretical contribution.

### 5.2 Recommendations for future studies and practitioners

While this study demonstrates the moderating role of gender in the relationship between physical activity enjoyment and mental well-being, future studies should adopt different methodological approaches to develop a more holistic perspective. Longitudinal studies, in particular, will provide a more robust analysis of the direction and dynamics of change in these relationships over time. In addition, future research should include samples from different universities and regions to allow for comparative analyses across diverse socio-cultural contexts, thereby enhancing the generalizability of the findings. Furthermore, gender should be considered not solely as a biological category but rather in conjunction with gender roles, identities, and their cognitive and psychological implications. Furthermore, comparative studies examining the impact of cultural differences will contribute to a contextual understanding of the psychological effects of physical activity on individuals. Quantitative findings can be more comprehensively supported by in-depth examinations of individuals’ physical activity experiences through qualitative or mixed-method research.

Various institutions, especially universities, should consider gender-based differences when planning physical activity programs. Because men and women derive different satisfaction from physical activity, the content and presentation of the activities offered should be diversified. Considering the emotional and social bonding orientation of females, aesthetic, group-based and/or relaxing activities can be more encouraged. For men, programs focused on competition, performance, and physical strength can increase enjoyment. Furthermore, participant-centered physical activity environments should be created to support students’ intrinsic motivation, and structures that meet individuals’ needs for autonomy, competence, and relatedness should be prioritized. In this context, programs developed based on SDT will be more effective in supporting both the physical and mental well-being of students.

## Data Availability

The raw data supporting the conclusions of this article will be made available by the authors without undue reservation.
